# A Point System for Predicting 10-Year Risk of Developing Type 2 Diabetes Mellitus in Japanese Men: Aichi Workers’ Cohort Study

**DOI:** 10.2188/jea.JE20170048

**Published:** 2018-08-05

**Authors:** Hiroshi Yatsuya, Yuanying Li, Yoshihisa Hirakawa, Atsuhiko Ota, Masaaki Matsunaga, Hilawe Esayas Haregot, Chifa Chiang, Yan Zhang, Koji Tamakoshi, Hideaki Toyoshima, Atsuko Aoyama

**Affiliations:** 1 Department of Public Health, Fujita Health University School of Medicine, Aichi, Japan; 2 Department of Public Health and Health Systems, Nagoya University Graduate School of Medicine, Aichi, Japan; 3 Department of Nursing, Nagoya University School of Health Science, Aichi, Japan; 4 Education and Clinical Research Training Center, Anjo Kosei Hospital, Aichi, Japan

**Keywords:** type 2 diabetes mellitus, incidence, risk prediction, cohort study

## Abstract

**Background:**

Relatively little evidence exists for type 2 diabetes mellitus (T2DM) prediction models from long-term follow-up studies in East Asians. This study aims to develop a point-based prediction model for 10-year risk of developing T2DM in middle-aged Japanese men.

**Methods:**

We followed 3,540 male participants of Aichi Workers’ Cohort Study, who were aged 35–64 years and were free of diabetes in 2002, until March 31, 2015. Baseline age, body mass index (BMI), smoking status, alcohol consumption, regular exercise, medication for dyslipidemia, diabetes family history, and blood levels of triglycerides (TG), high density lipoprotein cholesterol (HDLC) and fasting blood glucose (FBG) were examined using Cox proportional hazard model. Variables significantly associated with T2DM in univariable models were simultaneously entered in a multivariable model for determination of the final model using backward variable selection. Performance of an existing T2DM model when applied to the current dataset was compared to that obtained in the present study’s model.

**Results:**

During the median follow-up of 12.2 years, 342 incident T2DM cases were documented. The prediction system using points assigned to age, BMI, smoking status, diabetes family history, and TG and FBG showed reasonable discrimination (c-index: 0.77) and goodness-of-fit (Hosmer-Lemeshow test, *P* = 0.22). The present model outperformed the previous one in the present subjects.

**Conclusion:**

The point system, once validated in the other populations, could be applied to middle-aged Japanese male workers to identify those at high risk of developing T2DM. In addition, further investigation is also required to examine whether the use of this system will reduce incidence.

## INTRODUCTION

The epidemic of diabetes is alarming worldwide.^1^ Although both high-risk and population approaches are needed to tackle the epidemic, health check-ups are often implemented in community, worksite, or clinical settings in order to identify those at high risk.^2^^–^^4^ However, it is not necessarily clear how to stratify individuals by disease risk. Although a number of type 2 diabetes mellitus (T2DM) prediction models have been reported,^5^^,^^6^ there is a scarcity of evidence in East Asians, especially from long follow-up studies. The prevalence and extent of risk factors related to T2DM have been suggested to vary among ethnicities,^7^^–^^9^ so it would be relevant to construct ethnicity-specific prediction model for T2DM. Among previous East Asian studies, some^10^^–^^13^ implemented logistic regression analysis without considering how predictors affect time to diabetes occurrence, and two studies^14^^,^^15^ did not adopt lifestyles. From the viewpoint of primordial prevention, a long-term prediction model that included behavioral variables identified via long-term survival analysis would have significant importance. Long-term prediction model of T2DM using survival analysis has not been examined much in East Asians.^16^^–^^18^ Therefore, the aim of the current study is to develop a point system to estimate 10-year risk of developing T2DM incidence in a middle-aged worksite-based male Japanese cohort, where health check-ups are conducted annually. The current study would add some to existing literature through the use of more recent data, more than 10-year follow-up time, and use of survival analysis. Moreover, we cross-validated the present model with an existing model^17^ by comparing their performances when applied to the present population.

## METHODS

### Population

The Aichi Workers’ Cohort Study is an ongoing study on diabetes and cardiovascular diseases. The worksite is a local government located in central Japan. A baseline questionnaire survey on lifestyles was administered in 2002 to 6,648 (men: 5,177, women: 1,471) Japanese civil servants aged 35–69 years in the worksite. Because of the small number of women who developed T2DM during the follow-up, we limited the present study to men. Of the 5,177 participants, 4,335 also provided written consent to provide worksite’s annual health check-up data, as well as their medical history information. We excluded subjects aged 65 years or older at baseline (*n* = 6) and those with prevalent diabetes, defined as self-reported medication use, baseline fasting blood glucose (FBG) level ≥126 mg/dL, or Hemoglobin A1c (HbA1c) ≥6.5% (United States National Glycohemoglobin Standardization Program method) (*n* = 448). Subjects with missing values in smoking status, alcohol drinking habit, regular exercise, family history of diabetes, weight or height, FBG, triglycerides (TG), high density lipoprotein cholesterol (HDLC) (*n* = 341) were also excluded, leaving 3,540 men for the current analysis. The study protocol was approved by the Ethics Review Committee of Nagoya University School of Medicine, Nagoya, Japan and Fujita Health University, Toyoake, Japan.

### Measurements

Weight (to the nearest 0.5 kg) and height (to the nearest 0.1 cm) were measured with the subjects in typical indoor clothing but without shoes. Body mass index (BMI) was calculated as weight (kg) divided by the square of height (m). Venous blood samples were drawn after the subjects fasted for eight hours or overnight. FBG, HDLC, and TG were investigated serologically. HbA1c were measured using high-performance liquid chromatographic and immunoassay methods.

### Definition of risk factors

Age was used as a continuous variable. BMI was classified into four groups (<23, 23–<25 [reference], 25–<27.5, or ≥27.5 kg/m^2^). Smoking status were dichotomized (non-current [reference] or current smoking) based on self-report. Regular exercise was defined as that of moderate or higher intensities, frequencies of three days or more per week, and duration of 30 minutes or more per time. Alcohol consumption was estimated from the number of days of alcohol drinking and the amount of alcohol drank per occasion, and it was grouped into four categories (0 [reference], <23, 23–<46, or ≥46 g/day). Family history of diabetes was defined as the positive history reported for the first degree’s relatives. Medication use for dyslipidemia is a self-reported dichotomized variable. Blood levels of HDLC, TG, and FBG were categorized as the following, respectively: ≥40 mg/dL [reference] or <40 mg/dL; <150 [reference] or ≥150 mg/dL; and <100 [reference], 100–<110, or 110–<126 mg/dL. Since the hazard ratio for BMI category of <23 kg/m^2^ was null, we collapsed that category with the reference one in the analysis (ie, <25 kg/m^2^). Due to more than half of individuals missing data on HbA1c at baseline, we were not able to assess this variable for the prediction model.

### Follow-up and ascertainment of incident T2DM

Subjects were followed until T2DM incidence, death, retirement, or March 31, 2015, whichever came first. However, the end of follow-up for the retiree who agreed to be contacted by post and remained free of incident T2DM was set as the date of the last contact before March 31, 2015. Next, although age of the retirement is usually 60 years, some retirees were reemployed on the basis of their own intention and kept participating in the worksite’s annual health check-up after the age of 60. Death during employment was ascertained thorough the worksite’s health care division, and that after retirement was informed from the pension division.

Incident T2DM was determined from serum FBG level that first exceeded 126 mg/dL or, if available, through HbA1c level that became 6.5% or over (using the United States National Glycohemoglobin Standardization Program method). HbA1c testing was provided only to the employees aged 40, 45, 50, and 55 years old until 2007, and to those with positive urinary glucose after 2008, in the annual health check-up. In addition, self-administrated questionnaire surveys were carried out approximately biennially between 2004 and 2014 about initiation of any diabetic medications, including insulin. We previously confirmed the accuracy of self-report to be 95% among those who had provided written consent for us to review their medical records through their physicians in charge.^19^

### Statistical analysis

Cox proportional hazard model was used. Variables showing significant association with T2DM incidence in the univariable model (*P* < 0.05) were simultaneously entered into a multivariable model. Backward selection procedure was used to determine the final model with significant (*P* < 0.10) predictors.

A point system was developed according to the method used in the Framingham Study.^20^ Namely, one point was defined to correspond to a risk associated with 5-year increase in age (B = 0.01522 * 5 = 0.076). This B was referred to as the base regression unit. In order to assign points to age, we first organized the continuous age variable into 5-year interval categories and set the 35–39-year category as the reference. The middle age of the reference age category (ie, 37 years) was subtracted from that of the other age categories, and the differences were multiplied by the beta coefficient of age (0.01522) in the final model. The products are considered to indicate risk increment for each age category from the reference one. For risks regarding the categorized predictors (BMI category, smoking status, family history, and categories of fasting blood glucose and triglycerides), the beta-coefficients represents the risk increment from reference categories of each variable, and they were divided by base regression unit to yield respective points. A total point score for each individual was calculated by summing up points for each variable, and the corresponding 10-year T2DM risk was obtained.

The accuracy of the point system was assessed using discrimination and goodness-of-fit. For discrimination, Kaplan-Meier estimate of time dependent area under receiver operating curve was used.^21^^–^^23^ It was referred to as c-index here. Similarly, time-dependent sensitivity and specificity were displayed for 18 selected cut-off points. The c-index can have values from 0.5 (no discriminative ability) to 1.0 (perfect discrimination; ie, the model can perfectly distinguish individuals who experienced the outcome from those who did not). The c-index values of <0.70, 0.70 to <0.80, and >0.80 are judged to have poor, acceptable, and excellent discrimination, respectively.^24^ The goodness-of-fit was assessed by comparing the observed and predicted number of events in deciles of predicted risk using Hosmer-Lemeshow test for survival data. Briefly, the predicted numbers of events in each decile were obtained as the sum of 1 − the predicted survival probability for each individual. The test statistic was assumed to follow the chi-square distribution with eight degrees of freedom.^25^

A non-invasive model was also developed and the model’s performance, as well as its improvement by inclusion of invasive variables, was evaluated.

Given the fact that T2DM incidence ascertained after retirement was only from disease history questionnaire, the status of which might be more advanced compared to incident cases ascertained using blood tests, we conducted sensitivity analysis restricting to subjects aged less than 60 years at baseline (*n* = 3,423) and censoring the observation once the age has reached 60 years.

A two-tailed *P* value <0.05 was considered to indicate statistical significance. All analyses were conducted using SAS for Windows, version 9.4 (SAS Institute, Cary, NC, USA).

### Cross-validation

We applied a risk score created in the Ibaraki Prefecture’s Study to the present dataset in order to compare performance of the risk models. Ibaraki Prefecture’s Study’s risk model was selected as a reference since it uses a set of variables same as the present one. Since the Ibaraki Prefecture’s Study included subjects aged 40 years or older, the present dataset was also restricted to the same age subjects (*n* = 2,981).

## RESULTS

The mean age at baseline was 47.8 years. The prevalence of overweight and current smoking was 24.2% and 34.2%, respectively (Table 1). Of 3,540 participants, 342 developed T2DM during median of 12.2-year follow-up. Of them, 78% were ascertained from blood test (52% by FBG ≥126 mg/dL, 18% by HbA1c ≥6.5%, and 8% by both measurements), and 22% by self-report only.

**Table 1.** Mean (standard deviation) or percentage of risk factors and univariable hazard ratios for type 2 diabetes incidence (*n* = 3,540), Aichi, Japan, 2002–2015

Risk factors	Mean or %	Univariable HR (95% CI)
Age, years	47.8 (7.0)	1.03 (1.02–1.05)
Body mass index, kg/m^2^		
<23.0	47.8	0.72 (0.55–0.95)
23.0–<25.0	28.1	1 (Reference)
25.0–<27.5	18.3	1.53 (1.14–2.05)
≥27.5	5.9	2.95 (2.09–4.17)
Smoking status		
Non-current	65.8	1 (Reference)
Current	34.2	1.48 (1.20–1.84)
Fasting blood glucose, mg/dL		
<100	77.8	1 (Reference)
100–<110	16.3	3.28 (2.57–4.19)
110–<126	5.9	7.67 (5.81–10.13)
Triglycerides, mg/dL		
<150	69.6	1 (Reference)
≥150	30.4	2.16 (1.75–2.67)
High-density lipoprotein cholesterol, mg/dL		
≥40	87.0	1 (Reference)
<40	13.1	1.17 (0.87–1.58)
History of dyslipidemia		
No	96.3	1 (Reference)
Yes	3.7	2.17 (1.44–3.25)
Regular exercise		
Yes	6.4	1 (Reference)
No	93.6	1.28 (0.79–1.09)
Alcohol consumption, g/day		
0	22.9	1 (Reference)
<23.0	39.1	1.07 (0.80–1.43)
23.0–<46.0	24.7	1.18 (0.86–1.62)
≥46.0	13.3	1.34 (0.93–1.91)
Family history		
No	86.2	1 (Reference)
Yes	13.8	1.97 (1.53–2.53)

CI, confidence interval; HR, hazard ratio.

In the univariable models, variables other than HDLC, alcohol consumption, and regular exercise were significantly associated with T2DM incidence.

In the multivariable Cox proportional hazard model with backward selection procedure, age, BMI, smoking status, family history of diabetes, and blood levels of TG and FBG were retained (Table 2). Points were assigned for each of these predictors, with the highest point of 24 assigned to FBG of 110–<126 mg/dL, followed by 14 point to FBG 100–<110 mg/dL and 12 point to BMI ≥27.5 kg/m^2^. Total points could range from 0 to 58. The points 20, 25, and 30 represent 10%, 15%, and 20% risks of developing T2DM in 10 years, respectively. The points 16 and 25 represent 78% and 91% specificity, with corresponding sensitivity of 64% and 38%, respectively (Table 3).

**Table 2.** Multivariable Cox regression coefficients (standard errors) of the final type 2 diabetes mellitus risk prediction model and point assigned to predictors, Aichi, Japan, 2002–2015

Predictors	β (standard error)	*P* value	Points
Age, years	0.01522 (0.00846)	0.07	
35–39			0
40–44			1
45–49			2
50–54			3
55–59			4
60–64			5
Body mass index, kg/m^2^			
<25.0	Reference		0
25.0–<27.5	0.41620 (0.13172)	0.002	5
≥27.5	0.91153 (0.16210)	<0.0001	12
Smoking status			
Non-current	Reference		0
Current	0.32193 (0.11109)	0.004	4
Family history			
No	Reference		0
Yes	0.55512 (0.12913)	<0.0001	7
Triglycerides, mg/dL			
<150	Reference		0
≥150	0.43289 (0.11460)	0.0002	6
Fasting blood glucose, mg/dL			
<100	Reference		0
100–<110	1.07431 (0.12631)	<0.0001	14
110–<126	1.84067 (0.14629)	<0.0001	24

Variables were selected by backward selection procedure.

**Table 3.** Predicted 10-year risk of diabetes, sensitivity, and specificity according to 18 cut-off points, Aichi Workers’ Cohort Study, 2002–2015

Total points	Predicted risk, %	Sensitivity, %	Specificity, %
2	2.7	94	25
4	3.1	90	34
6	3.6	90	40
7	3.9	85	52
9	4.5	78	61
11	5.2	76	64
13	6.1	70	70
14	6.5	66	76
16	7.6	64	78
18	8.8	58	82
20	10.1	53	85
21	10.9	50	86
23	12.6	43	89
25	14.5	38	91
27	16.6	37	93
28	17.8	28	95
30	20.0	25	96
32	23.3	21	97

**Figure 1.**
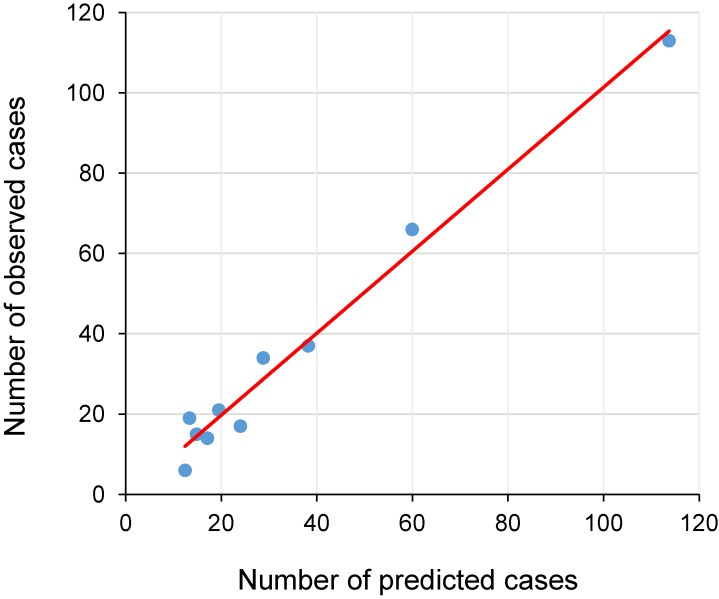
Goodness of fit plot showing predicted and observed number of incident type 2 diabetes mellitus according to deciles of predicted 10-year risk based on point system, Aichi, Japan, 2002–2015. Coefficient of the regression = 1.0210, constant of the regression = −0.6903. Solid point denotes each decile.

The point system discriminated incident T2DM reasonably well, with the c-index of 0.77. Also, the predicted number of incident cases of T2DM generally matched the numbers of observed cases according to the predicted 10-year risk deciles (Figure 1, Hosmer-Lemeshow test, *P* = 0.22). The discrimination for the non-invasive model that included age, BMI, smoking status, and family history of diabetes was 0.68, although the Hosmer-Lemeshow test was significant (*P* < 0.01). Further adding blood level of TG and FBG largely improved the performance, leading to a c-index of 0.77 and *P* value of 0.22 for the Hosmer-Lemeshow test (data now shown).

A sensitivity analysis restricted to subjects aged less than 60 years at baseline and censoring at the age of 60 years provided essentially similar findings.

The c-index when the Ibaraki Prefecture’s Study’s risk score was 0.72, lower than the present study c-index (0.77). The goodness-of-fit was also unsatisfactory (Hosmer-Lemeshow test *P* < 0.01) in contrast to the present model (*P* = 0.11).

## DISCUSSION

In the present study, we developed a reasonably well-calibrated and discriminative point system to predict 10-year T2DM risk in middle-aged Japanese using only six items: age, self-reported smoking and family history information, BMI, and blood levels of TG and FBG. These are easy to assess, widely available variables under the provision of health check-up. Participants scoring 30 points or more would be considered at high risk, with a more than 20% chance of developing T2DM. The cut-off point that maximizes the sum of sensitivity and specificity was 14 (sensitivity: 66%, specificity: 76%). A normoglycemic participant who currently smokes and whose BMI is in the range of 25.0–<27.5 kg/m^2^ and TG is ≥150 mg/dL would have equal or higher risk of developing T2DM compared to a subject with FBG of 100–<110 mg/dL with no other risk factors. We believe that our point system is useful to identify those at high risk and provide them options to change according to the individual’s preference or readiness in order to prevent or delay the onset of T2DM.

The rationale for our creation of another T2DM prediction model was somewhat suboptimal performance of an available model using similar variables (ie, the Ibaraki Prefecture Study) when applied to our dataset and possibly to the similar populations.

The variables selected in the present study were consistent with previous literature.^10^^–^^18^^,^^26^ Although screening of diabetes is recommended in asymptomatic patients with hypertension or cardiovascular disease, these variables did not improve predictability of the model in the present study. Since we additionally assumed that they would not be causal factors of diabetes, we did not include these variables in the present study. Although heavy alcohol drinking and physical activity are known to increase and decrease T2DM risks, respectively, they were not associated with T2DM in the present study even from the univariable models. Since it might be difficult to accurately measure these behaviors using questionnaires, we remain inconclusive regarding the association of these variables with T2DM. In addition, there is still a possibility that effects of these variables might be mediated by variables included in the present model.

A non-invasive model that could be used in a setting without laboratory information performed sub-optimally in the present study (compared to other studies). However, adding invasive variables (FBG and TG) significantly improved both discrimination and goodness-of-fit, which is compatible with the findings of previous reports.^11^^,^^18^ Although FBG categories were assigned high points in the present study, discrimination (c-index) of the glucose-only model was 0.68, much lower than that of the final model (0.77). Furthermore, goodness-of-fit of the glucose-only model was not adequate (Hosmer-Lemeshow test, *P* < 0.01). Our result would indicate that additional information other than blood measurements would be of value to predict long-term T2DM, especially in those having favorable clinical laboratory values.

One of the characteristics of the present study is the use of more recent data, the long-term follow-up, and use of survival analysis. A similarly long-term (ie, 10-year or more) prediction model of T2DM has not been examined much in East Asia^16^^–^^18^; we identified only two studies from Japanese^16^^,^^17^ and another from Taiwanese populations.^18^ Although existing studies, including ours, used similar or common predictors, some key variables are different. For example, we did not have data on waist circumference at baseline, which prevented us from cross-validating models from the Hisayama study^16^ and Taiwanese study^18^ using the present dataset. Although BMI and waist circumference are highly correlated and would provide similar information regarding the degree of adiposity at a population level, waist circumference is considered to be associated with adverse health outcomes more than BMI at an individual level. Further studies should be planned that include waist circumference data. In addition, the risk score of the Ibaraki Prefecture’s Study did not include family history, which might be an explanation for the inferior performance compared to the present risk model. Another strength of the present study is the identification of a sufficient number of T2DM cases, which enabled us to construct a multivariable model.

The c-index from our study is somewhat inferior to those reported from Western countries (0.79 and 0.85).^27^^,^^28^ We speculate that racial/ethnic differences in the pathophysiology of T2DM might be related to relatively worse prediction. That is, impaired insulin secretion is reportedly a more important characteristic of T2DM than insulin resistance among East Asians.^29^^,^^30^ However, most of the known risk factors of T2DM would be those related to insulin resistance.

Some limitations warrant consideration. First, our predictive model was derived from a worksite’s male employee. We were unable to validate the model in the same paper. The participants in the study may be a special group (civil servants), and the generalization to the other groups requires caution. For example, the present participants had lower prevalence of current smoking and overweight (BMI ≥25 kg/m^2^), but they also had lower prevalence of physical activity (two or more days per week for 30 minutes or more per occasion) at baseline compared to the 2002 National Health and Nutritional Survey of Japan. The incidence rate of T2DM could also be different, although it is difficult to compare among studies of different ages and different length of follow-up. External validation of the present model is needed before it can be applied in practice. Second, although HbA1c alone or combined with other risk factors has been shown to improve predictive performance,^12^^,^^13^ we could not assess the variable due to the limited number of participants with HbA1c at baseline. Inconsistent assessments of HbA1c and hypoglycemic medication use may raise biases and should be considered as another study limitation. Moreover, genetic information was unavailable in the present study. Although genetic risk equations have been attempted, most previous studies conducted in Western population did not appear to perform better than a non-genetic model.^31^^–^^33^ The issue, however, warrants further investigation in East Asian populations, which may have different genetic susceptibility to T2DM.^9^ In the present study, family history was included, which would be a composite of genetic and shared environmental factor among family members.^34^ Third, blood tests at fasting status (>8 hours) is necessary when applying the current point system, which might limit utilization at health promotion practices, like nationwide community health check-ups. In addition, diabetes was ascertained mainly by FBG and self-report, without undergoing an oral glucose tolerance test either at baseline or follow-up. It has been reported that T2DM identified by FBG alone may be underestimated.^35^^,^^36^ Hence, our prediction model may have performed better if T2DM had been diagnosed with other methods.

### Conclusion

In conclusion, we developed a point system in a middle-aged worksite-based male Japanese cohort. External validation of the system and intervention studies assessing the usefulness of the point system for the prevention of T2DM are warranted.
